# Management of Endometrial Intraepithelial Neoplasia: A Retrospective Review of Practice Patterns at a Single Military Treatment Facility and Civilian Partner Hospital

**DOI:** 10.1177/26884844251379404

**Published:** 2025-09-22

**Authors:** Kathleen R. Lundeberg, Rebecca W. Gregg, Erica R. Hope, Stuart S. Winkler, McKayla Riggs

**Affiliations:** ^1^ Department of Obstetrics and Gynecology, Wright State University Boonshoft School of Medicine, Dayton, OH, USA.; ^2^ Department of Gynecologic Surgery and Obstetrics, Brooke Army Medical Center, San Antonio, TX, USA.; ^3^ Department of Gynecologic Surgery and Obstetrics, Uniformed Services University of the Health Sciences, Bethesda, MD, USA.

**Keywords:** endometrial intraepithelial neoplasia, EIN, endometrial cancer, endometrial hyperplasia

## Abstract

**Background:**

Endometrial intraepithelial neoplasia (EIN) is a known precursor to endometrial endometrioid carcinoma, with a 40% risk of concurrent endometrial cancer at the time of hysterectomy. Many benign gynecologists (GYN) refer to gynecologic oncologists (GYO) for this risk of malignancy. This retrospective cohort study describes the pathological outcomes of patients with EIN who underwent hysterectomy at a single military treatment facility (MTF) and a civilian partner hospital.

**Methods:**

A retrospective chart review was performed for patients with a diagnosis of EIN at a single MTF and civilian hospital from 1/1/2021 to 10/30/2023. Preoperative risk factors for malignancy of cases performed by GYN and GYO were compared.

**Results:**

A total of 58 patients with EIN were included (8 from the MTF, 50 from the civilian partner hospital). Of these, 48 (82.8%) patients were referred to GYO for hysterectomy. Thirty-three (56.9%) patients were upstaged to endometrial cancer, with 28 (84.5%) Stage IA, 3 (9.1%) Stage IB, and 2 (3.4%) Stage III. Of the 48 patients in the GYO cohort, 36 patients underwent nodal sampling with only one lymph node positive for metastasis. No patients required secondary staging procedures. No significant preoperative risk factors were identified for advanced cancer.

**Conclusions:**

Our data supports prior literature suggesting that the likelihood of nodal involvement and advanced metastatic disease in patients with EIN is low. Current guidelines allow flexibility in the management of EIN by either GYN or GYO. Additional research into and application of preoperative risk algorithms may help identify patient risk for advanced malignancy and accurately guide low-risk patients towards management by GYN and high-risk patients by GYO.

## Introduction

Endometrial cancer is the leading gynecological cancer diagnosis in the United States, with endometrial intraepithelial neoplasia (EIN) as an established precursor lesion. Numerous risk factors have been identified for progression to endometrial cancer, including obesity, exposure to unopposed estrogen, nulliparity, anovulation, and genetic predisposition (*i.e.,* deficiency in mismatch repair proteins). Patients diagnosed with EIN have a 40–50% risk of concurrent endometrial cancer at the time of hysterectomy, with only 10% considered high risk for nodal involvement, which may require adjuvant therapy.^1^

The standard of care management for EIN is a total hysterectomy with or without a bilateral oophorectomy to both prevent progression to endometrial cancer and to detect underlying carcinoma. Sentinel lymph node (SLN) assessment at the time of hysterectomy for EIN is controversial, with multiple studies evaluating its cost-effectiveness while weighing the overall low risk of nodal involvement at the time of surgery. Before hysterectomy, the American College of Obstetricians & Gynecologists (ACOG) recommends that carcinoma be excluded using hysteroscopy and directed sampling for confirmatory endometrial.^2^ ACOG defers the decision of whether to consult GYO to the treating gynecologist based on the patient and health system’s available resources. In recent years, many of these patients have been referred to GYO for their surgical management. The primary concern with a GYN performing a hysterectomy for EIN is the inability to perform nodal dissection at the time of original surgery if indicated by intraoperative findings.

The reported estimated risk of nodal spread is 1.9% for EIN and 5% for endometrial carcinoma on final pathology.^3^ Multiple methods have been devised to triage which patients need nodal evaluation. One approach has been to utilize intraoperative pathological frozen analysis to determine the need for lymphadenectomy, often utilizing the Mayo Criteria.^3–4^ This allows for the omission of lymphadenectomy in patients where endometrial cancer is identified but deemed low risk for nodal metastasis based on grade, tumor size, and myometrial depth of invasion. An alternative approach has been to perform SLN mapping and dissection universally in EIN. Mueller et al. evaluated the role of SLN evaluation with a preoperative diagnosis of endometrial hyperplasia at a single institution. Of the patients reviewed, 44% had grade 1–2 endometrial cancer on final pathology, of which only 4% were stage 1B or higher.^5^ Of the patients without SLN evaluation, one patient (<6%) had stage II disease, and the remaining patients were upstaged on final pathology to stage IA. Of those with SLN evaluation, three patients (4%) were stage IB or greater on final pathology. They concluded that while SLN assessment may only detect a very small number of occult metastases, it provides staging information to guide adjuvant. The added staging information has the potential to de-escalate treatment from external beam radiation therapy to adjuvant brachytherapy alone in high intermediate-risk patients. Touhami et al. evaluated the efficacy of universal SLN dissection with universal lymphadenectomy of patients with EIN, with each patient serving as their own control.^6^ None (0/70) of the atypical hyperplasia (AH) patients had nodal metastases in contrast to the 6.25% (4/33) of patients with the preoperative AH-cannot-exclude-carcinoma group and cancer on final pathology.

A multivariate model reported by Dioun et al. demonstrated no significant increase in fixed costs for mapping compared with no nodal evaluation but noted an association with increased variable costs (costs that change depending on the volume of cases), with the mean cost of SLN dissection ranging from $9,520 to $9,975 for robotic-assisted and laparoscopic routes.^3^ Lim et al. performed a cost-effective analysis of hysterectomy with nodal analysis and noted an increased cost with universal sentinel node sampling with limited clinical benefit, recommending hysterectomy with frozen section and triaged nodal sampling as the continued standard of care.^7^ Work by Leite et al., however, assessed the benefit of nodal sampling in a community setting and found higher rates of endometrial carcinoma than expected, with nodal mapping not found to increase short-term complication rates or surgical times.^8^ Irrespective of if or how SLN evaluation should be performed, the decision to do so requires a GYO intraoperatively.

We present a retrospective observational cohort study comparing the pathology outcomes of patients with a preoperative diagnosis of EIN/CAH when performed by either a GYN or GYO at a single military treatment facility (MTF) and its civilian partner hospital. We hypothesize that the rates of our unique population comprised of military and non-military patients will reflect known national rates of concurrent endometrial cancer and advanced endometrial cancer in EIN patients and may provide additional background data in the eventual creation of EIN management guidelines within the greater military system.

## Methods

This research protocol was independently approved by the ethics committee of both institutions and Institutional Review Boards (IRBs). Two separate IRBs received exemption independently from the civilian and military hospitals for which retrospective data acquisition and review were performed (Wright State University with Data Use Agreement with Premier Health: 2023-419; Wright Patterson Medical Center: eIRB 946347). This study was conducted in compliance with the ethical standards of the responsible institution on human subjects. Patients included both military health beneficiaries at the MTF and civilian patients irrespective of insurance coverage at the civilian hospital.

Inclusion criteria required patients to be 18 years or older with a preoperative diagnosis of EIN or CAH based on pathology reports. Patients were managed with hysterectomy or conservative hormonal therapy at a single MTF and a single civilian partner hospital between 1/1/2021 and 10/1/2023. Diagnosis of EIN/CAH was made by endometrial biopsy, blind dilation and curettage, or directed sampling with hysteroscopy. Demographic, clinical, pathological, and surgical data were abstracted from the electronic medical records and reported with descriptive statistics using medians with ranges and percentages where appropriate. Race and ethnicity were documented to compare patient cohorts and were based on patients’ self-reported race or ethnicity. Preoperative documentation of endometrial thickness was reported in millimeters (mm). These variables were evaluated for statistical significance with paired t-tests or chi-square, with *p* ≤ 0.05 considered to be statistically significant. In accordance with the journal’s guidelines, we will provide our data for independent analysis by a team selected by the editorial team for additional data analysis or for the reproducibility of this study in other centers if such is requested.

## Results

A total of 58 patients underwent surgical management, and nine were medically managed. Demographics for the entire cohort included a mean age of 57.1, a BMI of 40.7, and an endometrial stripe of 14.1 mm (Table 1). Ten patients were surgically managed by a GYN and 48 by a GYO. The majority (86.2%) of patients self-identified as non-Hispanic White, 12.1% (7/58) as Black, and 1.7% (1/58) as Hispanic, and most patients denied a history of smoking. The demographic profile of patients at the MTF was comparable to that of the civilian cohort, with the majority of patients’ self-reported race/ethnicity as non-Hispanic White, with no significant difference in age or preoperative endometrial stripe thickness (EMS) (*p* = 0.46) (Table 2). A significant difference in BMI at the time of EIN diagnosis was noted between the MTF patients (BMI 32.7) and civilian patients (BMI 42.0) (Table 2, *p* < 0.01). When comparing the preoperative factors between GYN and GYO patients, there was no significant difference in age or preoperative endometrial stripe, but there was a significant difference in BMI (34.9 vs. 42.0, *p* = 0.029). The nine patients who were medically managed had a mean age of 48.6 (range 26–85, *p* = 0.29), BMI of 45.8 (range 24–92, *p* = 0.32), with five opting for fertility-sparing management and four deemed medically inoperable.

**Table 1.** Patient Demographics

Demographic	Total		GYN		GYO		*p* value
Age (years)	*N*	Mean (STD)	*N*	Mean (STD)	*N*	Mean (STD)	0.239
25–35	2	57.1 (±10.9)	0	54.9 (±13.9)	2	57.6 (±10.3)	
36–60	30		7		26		
>60	23		3		20		
Race/Ethnicity	*N*	% of total	*N*	% of total	*N*	% of total	<0.001^*^
White	50	86.2%	9	90%	41	85.4%	
Black	7	12.1%	1	10%	6	12.5%	
Hispanic	1	1.7%	—	—	1	2.1%	
BMI (kg/m^2^)	*N*	Mean (STD)	*N*	Mean (STD)	*N*	Mean (STD)	0.029^*^
<30	9	40.7 (±10.8)	3	34.9 (±12.3)	6	42.0 (±10.2)	
30–40	20		4		16		
>40	29		3		26		
EMS (mm)	*N*	Mean (STD)	*N*	Mean (STD)	*N*	Mean (STD)	0.246
2.0–9.9	14	14.09 (±6.1)	3	12.7 (±6.3)	11	14.4 (±6.1)	
10.0–20.0	24		4		20		
>20	9		1		8		
Tobacco	*N*	% of total	*N*	% of total	*N*	% of total	0.86
Yes	10	17.2%	3	30%	7	14.6%	
*N*o^*^	48	82.8%	7	70%	41	85.4%	

^*^ Denotes statistical significance with *p* <0.05.

**Table 2.** Military and Civilian Patient Demographic Profiles

Preoperative factor	MTF	Civilian	*p* value
Surgically managed			
Age (years)	56	57.3	0.38
BMI (kg/m^2^)	32.7	42	0.01^*^
EMS (mm)	13.8	14.1	0.46
Medically Managed			
Age (years)	42.7	51.5	0.29
BMI (kg/m^2^)	39	49.1	0.31
EMS (mm)^*^	26.7	8.6	0.07

^*^ Denotes statistical significance with *p* <0.05.

On final surgical pathology, 43.1% (25/58) were non-malignant (benign or EIN), and 56.9% (33/58) were malignant (Table 3, *p* = 0.29). Pathological outcomes by GYN versus GYO were compared (Table 3). Of the 10 patients managed by GYN, 5 were non-malignant on final pathology, and 5 were clinically staged as FIGO stage IA, grade 1 endometrial carcinoma. Of the 48 patients managed by GYO, 20 (41.7%) were non-malignant, and 28 (58.3%) were malignant on final pathology. This included 23 (82.1%) stage IA, 3 (20.7%) stage IB, and 2 (7.1%) stage III, all grade 1 endometrial carcinoma. There was no significant difference between the risk of malignancy on final pathology when comparing GYN and GYO (50% vs. 58.3%, *p* = 0.25).

**Table 3.** Final Pathological Outcomes

Final pathology	Total	GYN	GYO	*p* value
Benign/EIN	43.1% (25/58)	50% (5/10)	41.7% (20/48)	0.93
Stage IA	84.5% (28/33)	50% (5/10)	82.1% (23/28)	0.78
Stage IB	9.1% (3/33)	—	10.7% (3/28)	0.74
Stage III	6.1% (2/33)	—	7.14% (2/28)	0.79
Upstaged	56.9% (33/58)	50% (5/10)	58.3% (28/48)	0.94

The rate of advanced cancer (Stage III+) for this overall cohort was 3.4% (2/58). No secondary surgeries were required for adequate staging in the two patients diagnosed with advanced cancer on final pathology. Of the 48 patients surgically managed by GYO, 22 underwent SLN dissection and 23 utilized intraoperative frozen analysis to determine the risk for nodal involvement (14/23 proceeded with lymphadenectomy), and three had no assessment performed at the time of surgery. Of the 36 nodal analyses performed, only one (1/48) lymph node was positive for metastasis. Of those without nodal analysis were determined to be low risk based on intraoperative frozen sections (75%, 9/12), while the remaining had a preoperative plan for no nodal assessment at GYO discretion. Of those without any nodal assessment, 5/12 were found to have stage IA malignancy (41.7%) and underwent no further staging or adjuvant treatment. Of those that had nodal dissections, 61.1% had planned sentinel mapping, and 38.9% had lymph node assessment based on intraoperative frozen section.

Preoperative risk factors for patients with advanced malignancy were examined. One patient with advanced cancer on final pathology had a documented preoperative EMS of 19 mm, which was within one standard deviation of the mean (mean = 14 mm, STD ± 6.6 mm). While there was a difference in BMI between the MTF and civilian patients, there was no overall difference in BMI between the final pathological diagnosis of benign endometrium, EIN, and stage IA or stage II+ (Table 4). There was no significant difference in BMI among the stages in those with malignancy. There was a significant difference, however, in age and EMS thickness between the final pathology of non-malignant and stage IA, but this was not sustained at higher stages.

**Table 4.** Final Pathology and Preoperative Risk Factors

Preoperative demographic	Benign/EIN (*N* = 25)	Stage IA (*N* = 28)	Stage IB (*N* = 3)	Stage III (*N* = 2)	*p* value
Age (range)	53 (34–75)	60 (43–77)	62 (47–73)	58.5 (52,65)	0.01^*^ 0.11
BMI kg/m^2^ (range)	41.6 (19–66)	39.1 (20–61)	45.8 (47–56)	45.5 (36,55)	0.21 0.29
EMS (range)	11.8 (6–23)	15.9 (4–26)	12.96 (3–26)	19.1	0.01^*^ 0.38
Tobacco use^*^	20% (5/25)	14.3% (4/28)	—	—	0.5

^*^ Denotes statistical significance with *p* <0.05.

Two patients were noted to have advanced-stage (stage III) disease and were examined to identify additional preoperative risk factors (Table 5). Patient 1 had a BMI of 54, which was greater than the standard deviation for the average BMI for the civilian population (41.2 ± 9.1). Patient 2 had a rectosigmoid mass noted on preoperative imaging and concurrent active breast cancer, which led to her referral to a GYO for management.

**Table 5.** Patient Characteristics with Advanced-Stage Disease on Final Pathology

Stage III patients		
Preoperative factor	Patient 1: FIGO stage IIIC1	Patient 2: FIGO stage IIIB
MMR	Intact	Loss MLH1, PMS2
BRAF	Not reported	Negative
BMI	54	37
Tobacco use?	No	No
Concurrent primary cancer?	No	Yes

## Discussion

This study retrospectively compared the pathology outcomes of patients with EIN who underwent hysterectomy by a GYN versus a GYO at a single MTF and its local civilian partner. In our clinical setting, most patients who underwent hysterectomy for EIN were FIGO Stage IA (56.9%, Tables 1 and 2) and only 3.4% had advanced-stage disease (Stage II+). Our data reflects a lower risk of advanced disease than the reported 10% rate of advanced endometrial cancer at the time of hysterectomy.^1^ Of the two patients with advanced disease, one patient was referred to GYO management due to concurrent metastatic breast cancer with a pelvic mass, while the other was likely referred due to elevated BMI (54) and ease of accessibility to GYO services in this community.

On chart review, the local community practice pattern for most civilian cases revealed that a preoperative diagnosis of EIN reflexed a referral to GYO without further documentation of clinical decision-making based on identifiable risk factors. This may reflect the regional practice pattern of local GYNs in the civilian sector with readily available GYO surgeons. Furthermore, the GYO approach of nodal sampling at both the MTF and civilian partner hospital was nearly evenly split between SLN dissection and intraoperative frozen analysis (45.8% and 47.9%, respectively).

The Society of Gynecologic Oncology endorsed the ACOG Clinical Consensus on the Management of EIN, maintaining an individualized decision to refer to a GYO based on patient and system factors.^2,9^ Chaiken et al. found that patients undergoing hysterectomy for EIN had lower costs and higher quality-adjusted life years if performed by a GYO due to avoiding a secondary surgery for lymph node evaluation.^10^ Vetter et al. recommended GYO referral for patients with EIN if a preoperative endometrial stripe is ≥2 cm, because 44% of their patients diagnosed with endometrial cancer that met Mayo Criteria for lymphadenectomy were >2 cm, versus the 22% of patients with endometrial cancer with endometrial stripes <2 cm.^1^

Matanes et al. retrospectively assessed the outcomes of patients who underwent SLND at the time of hysterectomy for EIN and observed endometrial cancer for 37.7% of patients.^11^ Similar to our study, the majority of their patients had grade 1 and stage 1A disease (78.7% and 77.1%, respectively). There was no correlated increasing malignancy with BMI, age, and endometrial stripe.^11^ While they reported only two patients with stage IIIC disease (2/162, 1.2%), they identified a 9.2% risk avoidance for over- and undertreatment of patients who underwent SLND as part of their therapy, particularly those in the high-intermediate risk category.^11^

In our patient cohort, 21.4% with endometrial cancer had documented EMS thickness >2 cm and 75.0% with EMS ≤2 cm, while 5.0% (*n* = 1/20) with non-malignant final pathology had EMS thickness ≥2 cm. In this study, increasing age and endometrial stripe were noted to correlate with increased risk of malignancy (Table 4). This difference emphasizes the potential of utilizing preoperative risk factors, such as EMS, as a stratifying factor for GYO referral.

Tricare serves as a model of universal access to health care, with beneficiaries often facing fewer financial barriers to care. The military population is different from its civilian counterpart, with a statistically lower BMI and increased diversity. The military is also unique in that many MTFs are in geographically remote areas, and certain cases may require international transport of the member to another MTF for appropriate treatment—which can become a high financial cost; thus, appropriate triage of these patients is essential. While no data clearly reports the average cost of transport for health care in the Department of Defense, considering the cost of transport, housing, and the surgery itself—which is reported to range between $7,600 and $13,000—the total costs rapidly increase if patients are moved from a remote base to an MTF with GYO capability to undergo hysterectomy.^12^ The distribution of civilian GYOs across the US is centralized, with GYOs operating in only 8.8% of US counties and only 13.6% of GYOs traveling >50 miles to serve outlying hospitals.^13,14^ If safe and feasible, the utilization of GYN physicians to serve these populations could be beneficial for patient access to care.

Limitations of this study include the retrospective nature of the study, the small patient population from both the MTF and civilian hospitals, and patient selection *via* pathology reports. To combat this limitation, a secondary data retrieval was performed to ensure the validity of the process in obtaining patient reports. Our data further support the reported low risk for lymph nodal involvement and advanced malignancy in patients with an EIN diagnosis.

While gynecological malignancy data in the military population is not well reported, future directions include utilizing available data to create guidelines for active-duty military patients and Tricare beneficiaries diagnosed with EIN, especially when stationed in resource-limited areas remote from a GYO, balancing the appropriate standard of care with the economic cost. Current guidelines allow flexibility in the management of EIN by either GYN or GYO, additional research into and application of preoperative risk algorithms may help identify patient risk for advanced malignancy and accurately direct low-risk patients towards management by GYN and high-risk patients towards management by GYO. In the era of rising obesity rates and endometrial cancer diagnoses, this study emphasizes the ongoing need for strategic surgical planning for these patients.

## IRB Information

Wright State University with Data Use Agreement with Premier Health: 2023-419. Wright Patterson Medical Center: eIRB 946347.

## Abbreviations Used

ACOG: American College of Obstetricians & Gynecologists
BMI: body mass index
CAH: complex atypical hyperplasia
EIN: endometrial intraepithelial neoplasia
EMS: endometrial stripe thickness
GYN: gynecologist
GYO: gynecologic oncologist
MTF: military treatment facility
SLN: sentinel lymph node
